# Supplementary figures and images for: Transcriptomic Profiling of Cold Stress-Induced Differentially Expressed Genes in Seedling Stage of Indica Rice
Source: Plants (Basel). 2023 Jul 17;12(14):2675. doi: 10.3390/plants12142675 (PMC10384097; doi:10.3390/plants12142675)

## Slide 1
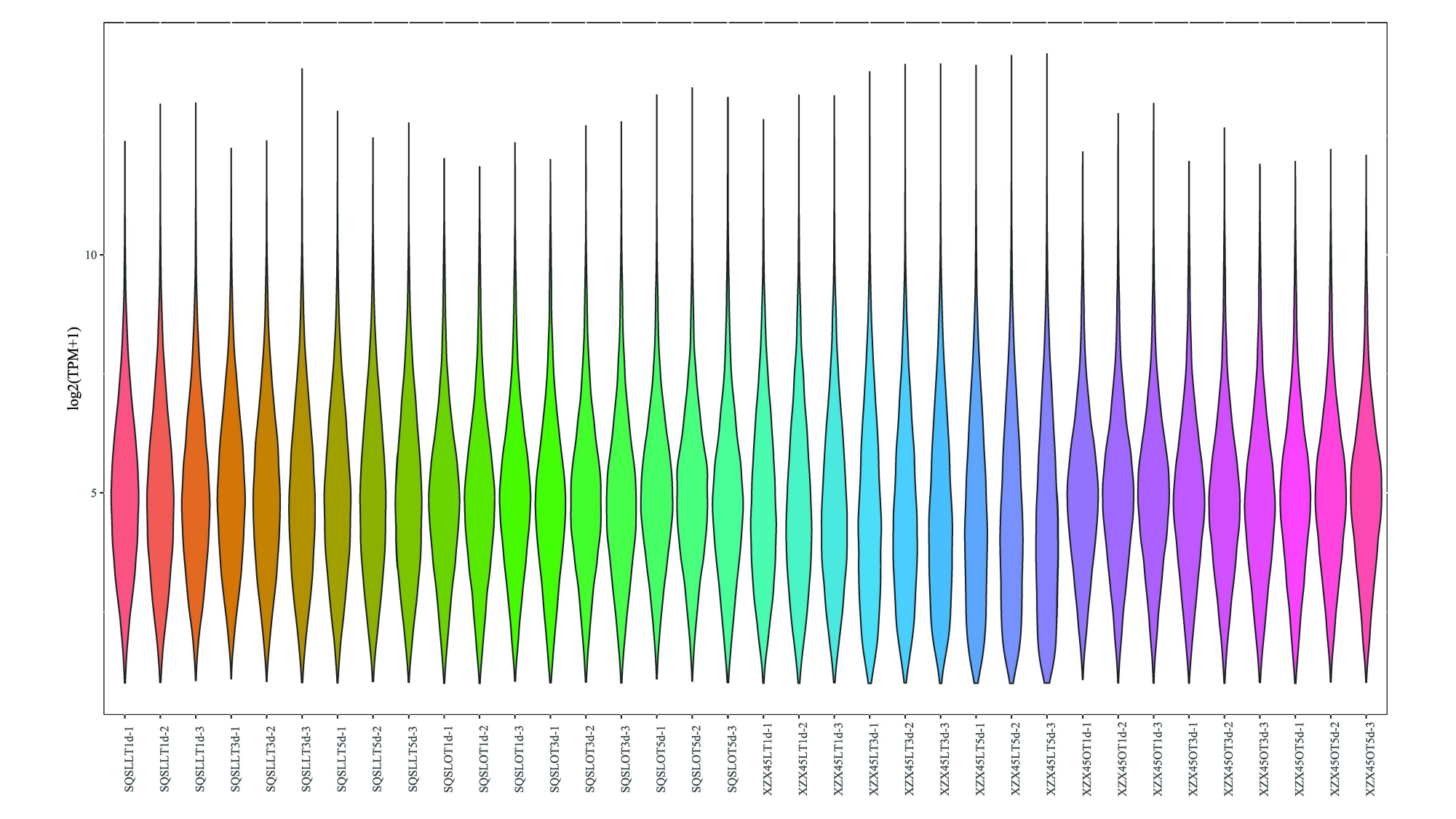

## Slide 2
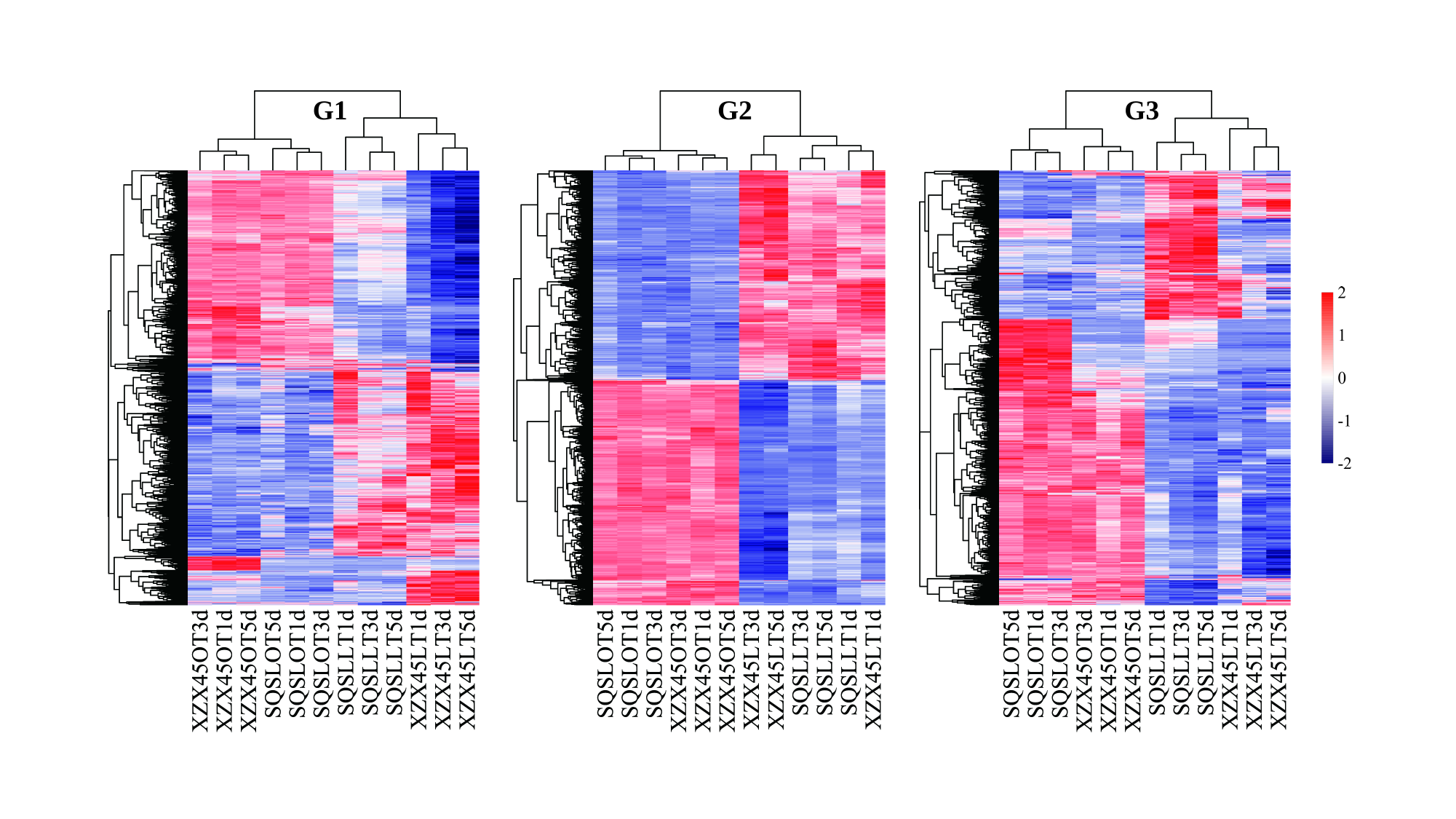

G1
G2
G3

## Slide 3
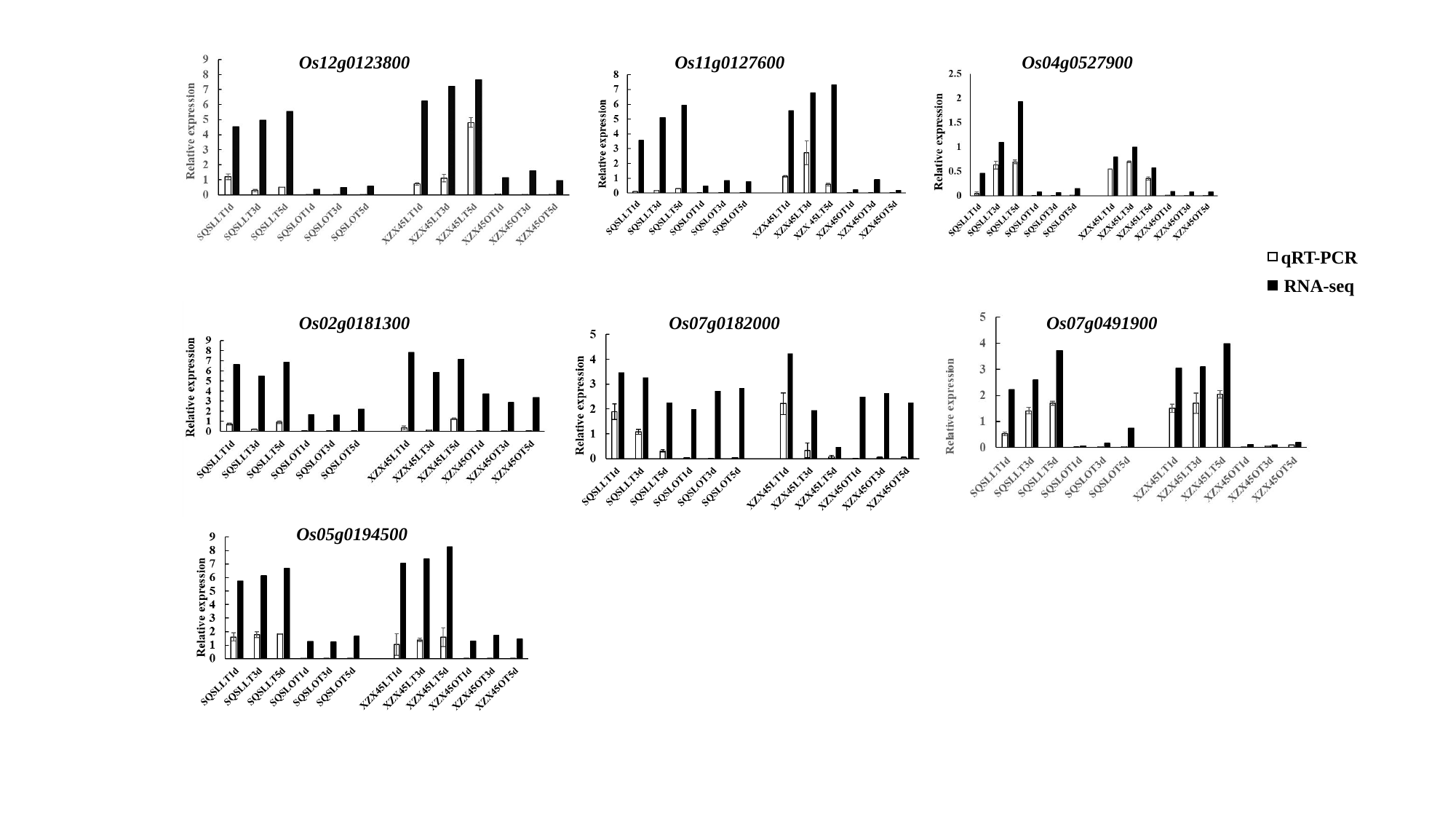

Os11g0127600
Os04g0527900
Os12g0123800
qRT-PCR
RNA-seq
Os02g0181300
Os07g0182000
Os07g0491900
Os05g0194500

Supplement: Supplementary file 1 [file plants-12-02675-s001.zip › plants-2488210.supplementary/Supplementary figures.pptx]
